# Role of Two-Component System Response Regulator *bceR* in the Antimicrobial Resistance, Virulence, Biofilm Formation, and Stress Response of Group B Streptococcus

**DOI:** 10.3389/fmicb.2019.00010

**Published:** 2019-01-23

**Authors:** Ying Yang, Mingjing Luo, Haokui Zhou, Carmen Li, Alison Luk, GuoPing Zhao, Kitty Fung, Margaret Ip

**Affiliations:** Department of Microbiology, The Chinese University of Hong Kong, Shatin, Hong Kong

**Keywords:** Group B Streptococcus, infection, two component system, *bceR*, antimicrobial peptide resistance, virulence, stress response

## Abstract

Group B Streptococcus (GBS; *Streptococcus agalactiae*) is a leading cause of sepsis in neonates and pregnant mothers worldwide. Whereas the hyper-virulent serogroup III clonal cluster 17 has been associated with neonatal disease and meningitis, serogroup III ST283 was recently implicated in invasive disease among non-pregnant adults in Asia. Here, through comparative genome analyses of invasive and non-invasive ST283 strains, we identified a truncated DNA-binding regulator of a two-component system in a non-invasive strain that was homologous to *Bacillus subtilis bceR*, encoding the *bceRSAB* response regulator, which was conserved among GBS strains. Using isogenic knockout and complementation mutants of the ST283 strain, we demonstrated that resistance to bacitracin and the human antimicrobial peptide cathelicidin LL-37 was reduced in the Δ*bceR* strain with MICs changing from 64 and 256 μg/ml to 0.25 and 64 μg/ml, respectively. Further, the ATP-binding cassette transporter was upregulated by sub-inhibitory concentrations of bacitracin in the wild-type strain. Upregulation of *dltA* in the wild-type strain was also observed and thought to explain the increased resistance to antimicrobial peptides. DltA, an enzyme involved in D-alanylation during the synthesis of wall teichoic acids, which mediates reduced antimicrobial susceptibility, was previously shown to be regulated by the *bceR*-type regulator in *Staphylococcus aureus.* In a murine infection model, we found that the Δ*bceR* mutation significantly reduced the mortality rate compared to that with the wild-type strain (*p* < 0.01). Moreover, this mutant was more susceptible to oxidative stress compared to the wild-type strain (*p* < 0.001) and was associated with reduced biofilm formation (*p* < 0.0001). Based on 2-DGE and mass spectrometry, we showed that downregulation of alkyl hydroperoxide reductase (AhpC), a Gls24 family stress protein, and alcohol dehydrogenase (Adh) in the Δ*bceR* strain might explain the attenuated virulence and compromised stress response. Together, we showed for the first time that the *bceR* regulator in GBS plays an important role in bacitracin and antimicrobial peptide resistance, virulence, survival under oxidative stress, and biofilm formation.

## Introduction

Group B Streptococcus (GBS) is the leading cause of sepsis in neonates and pregnant mothers worldwide (Russell et al., 2017; Seale et al., 2017). In particular, serogroup III sequence type (ST) 17 has been strongly associated with hyper-virulence as it causes neonatal sepsis and meningitis (D’Urzo et al., 2014; Seale et al., 2016). Further, life-threatening conditions associated with toxic shock syndrome and meningitis due to GBS are being increasingly reported in non-pregnant adults (Ballard et al., 2016). As in other regions, serotypes I, III, and V are predominant in invasive diseases of adults caused by GBS in Hong Kong (Skoff et al., 2009).

Group B Streptococcus serotype III-4/ST283 strains have been implicated in invasive diseases in non-pregnant adults in Asia (Wilder et al., 2000; Chan et al., 2002; Ip et al., 2006, 2016; Kalimuddin et al., 2017). Moreover, this ST283 type has been recently associated with an outbreak of invasive disease in adults in Singapore, which was suspected to be caused by the foodborne ingestion of contaminated freshwater fish as sushi (Kalimuddin et al., 2017). Compared to other serotypes identified in non-pregnant adults, GBS serotype III-4 has a significantly higher propensity to cause meningitis and septicemia, accounting for greater than 50% of all GBS meningitis cases in non-pregnant adults due to serotype III during 1993–2012 in Hong Kong (Ip et al., 2016). In Singapore, an outbreak of this strain type led to invasive diseases associated with spinal infection and septic arthritis in hundreds of young adults (Kalimuddin et al., 2017). Further, over the last 15 years, GBS serotype III-4 strains have remained a single clone of ST283, possessing distinct surface protein genes and mobile genetic elements and exhibiting indistinguishable PFGE fingerprints (Ip et al., 2006), suggesting that GBS III-4 strains might be hyper-virulent and possess special genetic virulent determinants.

Complete GBS genomes available in a public database (Genbank^1^) previously revealed that GBS possesses many pathogenic islands encoding virulence genes and transcriptional regulators, upon comparison with other streptococcal species (Glaser et al., 2002). Moreover, novel regulators involving two component systems (TCSs) associated with GBS pathogenesis have also been identified based on genome analyses (Samen et al., 2006, 2011; Lembo et al., 2010).

Two component systems are key bacterial regulatory systems involved in the detection and response to environmental challenges. Multiple TCSs have been reported in GBS, including covRS (Cumley et al., 2012; Sullivan et al., 2017), CsrRS (Park et al., 2012), RgfA (Al Safadi et al., 2011), and LtdR (Deng et al., 2018). These systems have been shown to play specific roles in colonization, pH tolerance during biofilm formation, and pathogenesis. In Gram-positive bacteria, many *bceR*-like systems have been characterized and comprise part of the antimicrobial peptide detoxification modules (Cui et al., 2005; Dintner et al., 2011). The best studied example of a *bceR*-like system is the bacitracin resistance module (*bceRSAB*) of *Bacillus subtilis* (Ohki et al., 2003; Cui et al., 2005). *In B. subtilis*, this system is linked to the ABC transporter, comprising the BceA ATPase and BceB permease, which serves as a detoxification pump for the removal of antimicrobial peptides (AMPs) (Ohki et al., 2003; Cui et al., 2005; Bernard et al., 2007). AMPs such as cathelicidins have an important role in mammalian innate immune defense and are produced by neutrophils, macrophages, and epithelial cells. However, Gram-positive bacteria have evolved resistance to these AMPs. Specifically, *Staphylococcus aureus* was reported to have two complete TCS/ABC transporter modules termed *graRS*-*vraFG* and *braRSAB* that either sense the same type of AMP or different AMPs and interact to mediate resistance (Cui et al., 2005; Li et al., 2007a; Meehl et al., 2007). In addition, *bceRS*-like systems such as *apsRS* in *S. epidermidis* and *graRS* in *S. aureus* not only enhance the expression of ABC transporters, but also lower the overall net negative charge of the cell envelope (Li et al., 2007b). This *aps* system decreases the anionic charge of the bacterial surface, which is specifically targeted by cationic AMPs (CAMPs), by upregulating the *dlt* operon and *mprF* (Li et al., 2007b). The *dlt* operon encodes proteins necessary for the D-alanylation of cell wall teichoic acid (TA), which through the repulsion of cations, confers resistance to AMPs (Peschel et al., 1999; Li et al., 2007b). In addition to AMP resistance, *graRS* of *S. aureus* was shown to play an important role in virulence, resistance to oxidative stress, and biofilm formation (Shanks et al., 2008; Falord et al., 2011).

In this work, we identified a key role for the response regulatory gene *bceR* in the determination of pathogenic traits in the clinically invasive GBS ST283 strain, including antimicrobial and oxidative stress resistance, biofilm formation, and virulence using a mouse infection model.

## Materials and Methods

### Bacterial Strains and Growth Conditions

Five GBS III-4 clinical strains were originally obtained from the Prince of Wales Hospital. The GBS strains selected for the current study were based on an archived collection of isolates from the Department of Microbiology, Chinese University of Hong Kong, Prince of Wales Hospital, and were previously characterized by molecular typing. The approval of clinical ethics for the laboratory typing of GBS strains with clinical demographics was obtained as a retrospective study (CRE-2012.054 from the Joint Chinese University of Hong Kong-New Territories East Cluster Clinical Research Ethics Committee) which was published (Ip et al., 2016).

The GBS strains were grown in Todd–Hewitt broth (THB) or THY broth (THB supplemented with 5 g/l yeast extract) or on THY blood agar plates (all from Difco Laboratories, Franklin Lakes, NJ, United States). Recombinant DNA manipulations were performed in *Escherichia coli* strain XL-Blue, grown at 37°C in Luria–Bertani (LB) broth (Difco Laboratories, Franklin Lakes, NJ, United States) or on LB agar plates.

### Whole Genome Sequencing and Comparative Genomics of Five GBS Serotype III-4 Strains

Five GBS strains of serotype III subtype 4 and sequence type ST283 were selected for genome sequencing (CU_GBS_00, CU_GBS_10, CU_GBS_12, CU_GBS_98, and CU_GBS_08). These strains were isolated in Hong Kong between 1998 and 2012, from both invasive and non-invasive sites in adult patients. Genomic DNA from the GBS strains was extracted using the Wizard^®^ Genomic DNA Purification Kit according to the manufacturer’s protocol for Gram-positive bacteria (Qiagen, Limburg, Netherlands). Genomes were assembled using the metAMOS pipeline (version 1.5rc3) (Koren et al., 2014). The draft genomes of CU_GBS_00, CU_GBS_10, and CU_GBS_12 were deposited in the NCBI database under GenBank accession numbers JYCT00000000, JYCU00000000, and JYCV00000000, respectively.

The genomes of CU_GBS_98 and CU_GBS_08 were completed (GenBank Accession numbers: CP010875 and CP010874, respectively). Draft genome scaffolds were built using the CONTIGuator software (version 2.7.4) (Galardini et al., 2011), with reference to a GBS complete genome (NEM316, accession number: NC_004368). Gaps between adjacent contigs were defined using Geneious (version R6.1.5^2^) and Mauve software (using progressive Mauve aligner, version 2.3.1; Darling et al., 2010). All gaps were successfully closed by PCR, and the complete genomes of CU_GBS_98 and CU_GBS_08 were deposited in the NCBI database.

We used MUMmer software (version 3.23; Kurtz et al., 2004) to align the GBS genomes to the complete reference genome of CU_GBS_08, to confirm the identified indels and SNPs. We used a cut-off value (breaklen = 500, distance to extend the genome alignment for poor scoring regions) to control for aligned regions considered by MUMmer for SNP and indel identification. The resulting genome alignments were also manually examined to identify gains or losses (and truncations) of genes that differed among GBS strains. Functional effects of the identified indels (in-frame or frame-shift indels) and SNPs (synonymous/non-synonymous/stop-codon mutations) were determined according to gene annotations based on the reference genome.

### Generation of Δ*bceR* Strain Using Allelic Replacement

The PCR products containing (a) ∼900 bp of sequence upstream from the *bceR* gene and (b) the last 58 bp of the *bceR* gene to approximately 900 bp downstream of the gene were amplified by PCR (Supplementary Table S1). The fragments were digested by the restriction enzyme *EcoRI* and ligated with T4 DNA ligase according to the manufacturer’s protocol (NEB, MA, United States). The ligated products were amplified by crossover PCR. The PCR product and the thermosensitive plasmid pJRS233 (Ashbaugh et al., 1998) were digested with restriction enzymes *KpnI* and *BamHI*, ligated, and then transformed into XL1-Blue competent cells (Agilent, CA, United States). The resulting plasmid was extracted with the Plasmid Maxi Kit (Qiagen, Limburg, Netherlands) and transformed by electroporation into CU_GBS_08 (Framson et al., 1997). Transformants were selected at 30°C with 1 μg/ml erythromycin on Todd Hewitt agar with 0.5% yeast extract and 5% defibrinated horse blood. Cells with the plasmid integrated into the chromosome were selected at 37°C under erythromycin pressure, and subsequently passaged at the same temperature in the absence of erythromycin for plasmid excision.

### Construction of Complementation Plasmid to Rescue Δ*bceR* Phenotypes

A plasmid was constructed to express full-length *bceR*, and a 500-bp fragment of the upstream region of this gene was amplified with primers containing *BamHI* and *Xbal* sites and cloned into the *BamH*I and *Xbal* sites of pDL289 (Soualhine et al., 2005) to create the *bceR* expression vector pDL289-bceR. Inserts and reading frames were confirmed by sequencing. pDL289-*bceR* was introduced into the Δ*bceR* strain by electroporation.

### Minimum Inhibitory Concentration (MIC) Determination

The MIC of antimicrobial agents was determined by the microbroth dilution method, according to the Clinical and Laboratory Standards Institute (CLSI, 2011).

### RNA Extraction and Real Time-PCR

The GBS was plated on blood agar plates and incubated at 35°C in 5% CO_2_. Sub-inhibitory bacitracin concentration values were determined by monitoring cell growth in THB with or without a range of bacitracin concentrations in 96-well plates. In brief, overnight cultures of cells were resuspended and adjusted to an OD_600_ of 0.8. A 1% bacterial suspension was prepared to obtain a final inoculum of 1 × 10^6^ to 5 × 10^6^ CFU per well in 200 μl of THB with or without bacitracin at 1/2, 1/4, and 1/8× the MICs. The bacterial cells were then incubated at 37°C, and the OD_595_ was measured every 30 min using a DTX 880 microplate reader (Molecular Devices, San Jose, CA, United States) over 24 h. The minimum concentration that did not alter the bacterial growth curve was considered the sub-inhibitory concentration for the described experiment. Experiments were repeated in triplicate.

Briefly, 2 ml of cultures was harvested at mid-log phase and cells were pelleted by centrifugation at 6000 × *g* at 4°C for 10 min. The pellets were resuspended in TE buffer containing RNA protect (Qiagen, Hilden, Germany) at a ratio of 1:2 TE:RNA protect for RNA stabilization. The bacterial suspension was then incubated with 400 μl of lysozyme (prepared in TE buffer) (Sigma, MO, United States) at 37°C for 30 min. The lysate was treated with 30 μl of 3 M sodium acetate (Sigma, MO, United States), 90 μl of 10% SDS (Merck, Gernsheim, Germany), and 1 ml of Trizol (Life Technologies, Camarillo, CA, United States). This was followed by a 5-min incubation at RT before adding 200 μl of chloroform (Merck, Gernsheim, Germany) for 2 min. All samples were centrifuged at 12,000 × *g* at 4°C for 15 min. The supernatant was transferred to a new tube with 1 ml of isopropanol (Merck, Gernsheim, Germany) for RNA precipitation. After 2 h of incubation at -20°C, the tubes were centrifuged at 12,000 × *g* at 4°C for 15 min and the supernatant discarded. An equal volume of cold absolute ethanol (Merck, Gernsheim Germany) was then added to the tube, which was centrifuged at 12,000 × *g* at 4°C for 5 min to obtain the RNA pellet. The pellet was resuspended in 100 μl of DNase-free and RNase-free water. Additionally, the sample was treated with 2 U of DNase I (Promega, Fitchburg, WI, United States) followed by a 20-min incubation at 37°C. The RNA quality and quantity were determine using a Nanodrop 1000 (Life Technologies, Camarillo, CA, United States), and the sample was then stored in 20-μl aliquots at -80°C.

Total RNA was extracted with Trizol (Chomczynski and Sacchi, 2006) for three independent experiments. Briefly, 200 ng of total RNA for each sample was subjected to cDNA synthesis using a TURBO DNA-free Kit (Thermo Fisher, MA, United States) according to the manufacturer’s protocol. The DNase inactivation reagent was removed by centrifugation at 10,000 × *g* for 1.5 min and the supernatant was aliquoted into fresh tubes for the reverse transcription step using SuperScript III Reverse Transcriptase (Invitrogen, CA, United States) according to the manufacturer’s protocol. Real-time PCR was performed using SYBR Green PCR Master Mix (Invitrogen, CA, United States) based on the manufacturer’s instructions, with an ABi 7500 Real-Time PCR Detection System (Applied Biosystems, MA, United States). Each sample was run in triplicate with 300 nM of each primer (Supplementary Table S2) with the following conditions: 95°C for 10 min, 40 cycles of 95°C for 30 s, and then 60°C for 1 min. Melting curves were generated by a cycle of 95°C for 1 min and 60°C for 1 min. The relative quantitation of mRNA expression was normalized to the constitutive expression of the 16S rRNA housekeeping gene and calculated by the comparative ΔΔCT method (Livak and Schmittgen, 2001; Wang et al., 2014).

### Mitogenicity and Cytokine Release in Human Lymphocytes

Bacteria were grown in THB (Oxoid) with 0.2% yeast extract overnight at 37°C. The overnight cultures were then diluted 1:100 in fresh THB, grown to mid-log phase, harvested by centrifugation at 3000 × *g* for 10 min, and then washed three times with phosphate-buffered saline (PBS). Pelleted cells were resuspended in PBS, heat-killed (100°C, 30 min), and subjected to centrifugation at 11,000 × *g* for 20 min at 4°C to remove cell debris. The supernatant (GBS cell extract) was aliquoted and stored at -80°C until required. Protein concentrations were determined using protein assay dye reagent concentrate (Bio-Rad) with bovine serum albumin (Sigma) as a standard.

Peripheral blood mononuclear cells (PBMCs) were isolated from the whole blood of healthy individuals (obtained from the Hong Kong Red Cross Blood Transfusion Service) by density gradient centrifugation using Ficoll-Paque (GE Healthcare). The human mononuclear cells were washed with PBS, resuspended in medium (RPMI 1640 with 10% FBS), and seeded at 2 × 10^5^ per ml in a 96-well View Plate (Perkin Elmer). Twenty-four hours later, GBS cell extract (prepared as described in the bacterial strains and growth conditions sections) was added at a final concentration of 25 μg/ml. Phytohemagglutinin (PHA, 10 μg/ml) and culture medium alone were included as controls. After incubation for 24 h, the proliferation of lymphocytes was detected using alamarBlue (Life Technologies) according to the manufacturer’s protocol. Fluorescence emission was measured using an EnSpire Multimode Plate Reader (Perkin Elmer) at 585 nm with an excitation wavelength of 570 nm. Experiments were performed in triplicate.

### Cytokine Measurements

After stimulating PBMCs, the supernatant from cell cultures was collected after incubation for 3, 6, 12, and 24 h to measure cytokine release. Interleukin (IL)-1β, IL-6, IL-8, IL-10, IL-12, and tumor necrosis factor alpha (TNF-α) were evaluated by ELISA according to the manufacturer’s instructions (BD Biosciences). Measurements were performed at an OD of 450 nm (EnSpire Multimode Plate Readers, PerkinElmer).

### Mouse Infection Model

Animal experiments were performed with permission of the Animal Experimentation Ethics Committee (AEEC) of the Chinese University of Hong Kong.

The virulence of Δ*bceR* GBS III-4 mutant strains was compared to that of the wild-type strain CU_GBS_08, the CU_GBS_12 strain with a natural truncation of *bceR*, and the ATCC 12403 Type strain as a control using a mouse model. The ATCC strain belongs to serogroup III and originated from a case of fatal septicemia^3^. The GBS inoculum was prepared by diluting overnight cultures 1:100 into THB. Cultures were incubated at 35°C, and then bacteria were harvested by centrifugation at 1200 × *g* for 10 min at 4°C. The pellet was then washed twice and resuspended in 5 ml of PBS. GBS was then prepared by diluting the PBS suspension to 10^7^ CFU/ml. Dilutions were confirmed by colony counts on blood agar. Six-week-old CD1 mice were purchased from The Laboratory Animal Services Centre (The Chinese University of Hong Kong, Hong Kong) and infected via intraperitoneal injection with 0.1 ml of the GBS inoculum at 10^7^ CFU/ml. The control group was injected with an equivalent volume of sterile PBS. Each group contained 30 mice. The mice were monitored for 10 days and those surviving at this time were sacrificed under anesthesia. The health condition of the mice was monitored daily and animals showing signs of excess weight loss, severe pain, and distress were euthanized before the end of study. The LD_50_ was calculated, and the Kaplan–Meier survival curve for infection and control groups with an endpoint of 10 days was prepared. The study was approved by the University Animal Experimentation Ethics Committee (AEEC; Reference no.:13-063-MIS) and conducted at The Laboratory Animal Services Centre in compliance with International Guiding Principles for Biomedical Research Involving Animals and The Hong Kong Code of Practice for Care and Use of Animals for Experimental Purposes.

### H_2_O_2_ Stress Assay

The GBS strains were plated on blood agar plates and incubated at 35°C in 5% CO_2_. Bacterial cells were suspended in pre-warmed THB with shaking at 200 rpm overnight. The overnight cultured bacterial cells were then diluted 1:100 in THB and incubated at 37°C with shaking at 200 rpm to achieve an OD_600_ of 0.8–1.0. The bacteria were resuspended in THB at a concentration of 4 × 10^7^ CFU/ml, and then 40 mM H_2_O_2_ was added at RT for 15 min. After treatment, fresh THY broth was added to stop the reaction and the bacteria were harvested by centrifugation at 4000 × *g* for 15 min. Bacterial viability after H_2_O_2_ treatment was then examined through the culture and enumeration of bacterial colonies. Serial dilutions of medium were used for CFU counting. Each experiment was conducted in triplicate.

### Determination of Biofilm Biomass by Crystal Violet Staining and CFU Counting

The GBS strains were plated on blood agar plates and incubated at 35°C in 5% CO_2_. Overnight bacterial cultures were then suspended in pre-warmed THB overnight and 24-well flat bottom plates (Costar, Boston, MA, United States) were used to support biofilm growth. Then, the overnight bacterial cultures were diluted 1:100 in THB and incubated at 37°C with shaking at 200 rpm to achieve an OD_600_ of 0.8–1.0. The bacteria were harvested by centrifugation at 4000 × *g* for 15 min. After washing with PBS, the cells were diluted 1:10 with pre-warmed THB, and 500 μl of cells was added to each well of a 24-well plate and incubated at 37°C with 5% CO_2_ overnight without shaking. All samples were run in triplicate.

Biofilm biomass was quantified by measuring the absorbance of crystal violet (Olson et al., 2002). After removing the culture medium, the plates were gently washed with PBS twice to remove the floating cells. Biofilms were stained with 300 μl of 0.5% crystal violet (Sigma, MO, United States) (prepared in 10% ethanol) for 15 min at RT. After staining, the plates were gently washed with PBS three times and dried at RT. Then, 500 μl of 95% ethanol was added to each well and incubated for 15 min to dissolve the biofilms. OD_595_ values were measured using a DTX 880 plate reader (Molecular Devices, San Jose, CA, United States).

Bacterial viability in biofilms was also examined by enumerating bacterial colonies. After removing the culture medium, the plates were gently washed with PBS twice to remove floating cells, which was followed by the addition of 500 μl of fresh THB to each well. The cells were collected by scraping the bottom of each well with a sterile cell scraper. Serial dilutions of the medium were used for CFU enumeration, and each experiment was performed in triplicate.

### Two-Dimensional Gel Electrophoresis (2DE) and Mass Spectrometry

The GBS strains were plated on blood agar plates and incubated at 35°C in 5% CO_2_. Bacterial cells were suspended in pre-warmed THB with shaking at 200 rpm overnight. Then, the overnight bacterial cultures were diluted1:100 in THB and incubated at 35°C with shaking at 200 rpm to mid-log phase, after which, the bacterial cells were harvested by centrifuging at 4000 × *g* for 20 min at 4°C. For whole protein extraction, the instructions of the total protein extraction kit (Bio-Rad, United States) were followed, and protein quantitation was performed using RC DC Protein Assay reagent (Bio-Rad, United States). Then, 2DE was conducted following the protocol of a previous study (Jones et al., 2004).

The gel photos were normalized and compared using software PDQuest (Version8.0.1, Bio-Rad, United States). The Boolean method was chosen to compare the intensity of the protein spots to determine both fold-changes and statistically significantly differences between GBS III-4 wild-type and Δ*bceR* strains. From the results, we found that the expression of three proteins was significantly decreased in the Δ*bceR* strain (>2-fold reduction in expression), and these three protein spots were cut from the original 2-DE gel and sent to the proteomic core laboratory of The University of Hong Kong for mass spectrometry-based identification.

### Statistical Analysis

Data are expressed as the mean ± SD. Statistical comparisons between different treatment groups were performed using a one-way analysis of variance (ANOVA), followed by a *post hoc* Dunnett’s test using GraphPad Prism 6.05 for Windows (GraphPad Software, San Diego CA, United States). Differences were considered as significant at *p* < 0.05, and were denoted as ^∗^*p* < 0.05, ^∗∗^*p* < 0.01, ^∗∗∗^*p* < 0.001, and ^∗∗∗∗^*p* < 0.0001.

## Results

### Whole Genome Sequencing and Comparative Genomics Analysis of GBS Serotype III-4 Strains

The genomes of three invasive and two non-invasive GBS serotype III-4 strains were sequenced using a Roche 454 and Illumina Solexa Genome Analyzer, according to the manufacturer’s instructions, and have been submitted to GenBank as either draft or complete genomes (Table 1). The genomes of the meningitis/septicemia strains were compared to those of the non-invasive strains. All single nucleotide polymorphisms (SNPs) from the ORFs were called using Mauve (version 2.3.1) software (Darling et al., 2010). Sequence alignment was performed to compare gene sequence variations among these strains. Genes that encode hypothetical proteins and those related to bacteriophages were not analyzed further. From this, we narrowed down our list to four truncated genes of interest as indicated in Supplementary Table S3. These genes showed 100% nucleotide identity to those of other GBS strains in GenBank. SNPs were confirmed by PCR-based Sanger sequencing to filter out false positive SNPs, which can occur with next generation sequencing.

**Table 1:** List of strains in this study.

GBS strain	GenBank accession number	Isolation site	Clinical details	Patient age group	Sequence type	Molecular serotyping group
ATCC12403 (NEM316)	NC_004368	Blood	Septicaemia	Infant	23	III
CU_GBS_00	JYCT00000000	Wound	Non-invasive	Non-pregnant adult	283	III-4
CU_GBS_08	CP010874	Blood	Toxic shock syndrome	Non-pregnant adult	283	III-4
CU_GBS_10	JYCU00000000	Blood	Septic arthritis	Non-pregnant adult	283	III-4
CU_GBS_12	JYCV00000000	Vaginal-rectal swab	Non-invasive	Pregnant adult	283	III-4
CU_GBS_98	CP010875	Cerebrospinal fluid	Meningitis	Non-pregnant adult	283	III-4
CU_GBS_08_Δ*bceR*	*bceR* deletion mutant of CU_GBS_08					
CU_GBS_08_Δ*bceR+pbceR*	*bceR complementation* of CU_GBS_08_Δ*bceR*					

**Figure 1:**
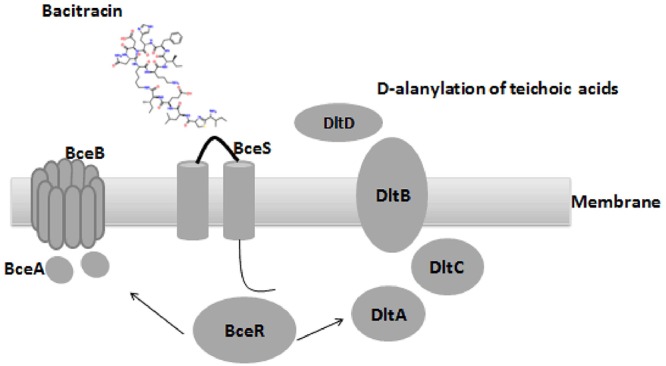
Proposed organization structure of BceRS system and working model in GBS. AMPs that usually function by pore formation in the membrane (bacitracin was used in this figure), bacitracin bind to the anionic loop of BceS and cause the phosphorylation of BceR, which result in the activation of BceR regulated AMPs resistance in GBS. (1) up-regulated the expression of the transporter BceAB to facilitate the resistance by expelling the bacitracin **(left)**. (2) D-alanylation of teichoic acids by the *dlt* system, which in turn decreased negative charge of the bacterial membrane and ensure the resistance **(right)**.

Comparative genome analysis revealed a non-synonymous substitution (truncation) of a DNA binding regulator (Accession no: CU_GBS08_01010) in the non-invasive GBS strain, and the truncation of *bceR* at c.288delG was determined to generate a stop codon, abrogating expression of a region of the mRNA encoding the last 20 aa of the receiver domain and the DNA-binding domain. BLAST analyses revealed that this regulator was most closely related to the TCS response regulator protein BceR of *S. gallolyticus*, with 69% protein sequence homology (GenBank no: CDO17747.1). Although this gene was present in all GBS strains examined, the sequences harbored ∼30% differences compared to the *bceR* genes of other bacteria, suggesting that this gene might have specific functions in GBS. Based on the location of the truncation of the response gene, we predicted that the *bceR*-like response would be aborted in the non-invasive strain. The present study therefore focused on the role of this response regulator gene in this TCS of GBS. We thus knocked down this gene in the wild-type invasive strain CU_GBS_08 to elucidate its role in antimicrobial peptide resistance, stress response, and virulence in this invasive GBS strain. Our working model is depicted in Figure 1. Together with evidence that the transporter-encoding *bceAB* gene is activated by bacitracin, we have re-named this regulator *bceR* of the two-component system *bceRS* in this complete genome (GenBank genome: CP010874).

### The Δ*bceR* Strain Is More Sensitive to Bacitracin and Antimicrobial Peptides

It is known that *bceR*-like systems comprise components of antimicrobial peptide detoxification modules, such as the *graRS* system of *S. aureus*, as the MIC values of some AMPs were decreased in strains with mutations in this system (Cui et al., 2005; Meehl et al., 2007). Here, the MICs of selected AMPs and antibiotics were measured for the Δ*bceR*, complementation, and wild-type strains (Table 2). MICs for the mutant strain were 256- and 4-fold lower for bacitracin and LL-37, respectively, compared to those for the wild-type strain. However, Δ*bceR* complementation with the pDL289-*bceR* plasmid restored resistance to both bacitracin and LL-37 (Table 2). No difference in resistance was observed between the wild-type strain and the isogenic Δ*bceR* strain for other antibiotics.

**Table 2:** Minimal inhibitory concentrations (MIC) of antimicrobial peptides and other antibiotics in GBS strains.

	MICs (μg/ml)^a^				
**Antibiotics**	**CU_GBS_08**	**CU_GBS_08_Δ*bceR***	**CU_GBS_08_Δ*bceR+pbceR***	**CU_GBS_12**	**ATCC49619**

Bacitracin	64	0.25	64	0.25	8
LL-37	256	64	256	128	2
Polymyxin B	128	128	128	64	128
Ampicillin	≤0.06	≤0.06	≤0.06	≤0.06	≤0.06
Cefotaxime	≤0.06	≤0.06	≤0.06	≤0.06	≤0.06
Penicillin	≤0.06	≤0.06	≤0.06	≤0.06	≤0.06
Vancomycin	0.5	0.5	0.5	0.5	0.5
Erythromycin	≤0.06	≤0.06	≤ 0.06	≤0.06	≤0.06
Ciprofloxacin	0.5	0.5	0.5	0.5	0.5

*^*a*^Minimum inhibitory concentration, obtained according to CLSI protocol (CLSI, 2011)*.

### Expression of *bceA*, *bceB*, and *dltA* Is Reduced in the Δ*bceR* GBS Strain

**Figure 2:**
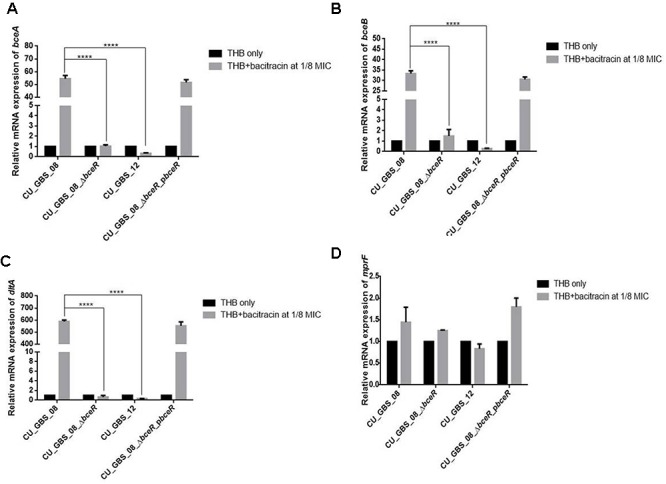
Relative expression of *bceA*, *bceB*, *dltA*, and *mprF* **(A–D)** in GBS strains with addition of bacitracin. Genes expression of *bceA*, *bceB*, *dltA*, and *mprF* in wild type GBS III-4 strain, CU_GBS_08 (bacitracin MIC 64 μg/ml), isogenic mutant (CU_GBS_08_Δ*bceR*, bacitracin MIC 0.25 μg/ml), and non-invasive GBS III-4 strain CU_GBS_12 (bacitracin MIC 0.25 μg/ml) were shown. GBS strains without treatment were normalized to 1. Error bars represent the standard deviation of the mean values from three independent experiments. Significance was determined by one-way ANOVA (^∗^*p* < 0.05, ^∗∗∗∗^*p* < 0.0001).

*graRS*, a *bceRS*-like system of *S. aureus*, was reported to induce AMP resistance not only by pumping AMPs out via an ABC transporter, but also by lowering the overall negative net charge of the cell envelope by upregulating expression of the *dlt* operon and *mprF* (Li et al., 2007; Meehl et al., 2007). Thus, the expression of *bceA, bceB*, *dltA*, and *mprF* was evaluated in the presence of a sub-inhibitory concentration of bacitracin in wild-type and Δ*bceR* strains and normalized to 16s rRNA expression. Expression levels in GBS strains grown in THB only were used as controls and adjusted to 1. As shown in Figure 2, levels of *bceA*, *bceB*, and *dltA* were higher when respective strains were grown in THB containing bacitracin at 1/8 the MIC value for CU_GBS_08 (bacitracin: MIC, 64 μg/ml) compared to those when bacteria were grown in the presence of bacitracin at 1/8 the MIC value for CU_GBS_Δ*bceR* (bacitracin: MIC, 0.25 μg/ml; *p* < 0.0001) and for CU_GBS_12 (bacitracin: MIC, 0.25 μg/ml; *p* < 0.0001; Figures 2A–C). However, no significant difference of *mprF* expression was found between the wild-type strain and Δ*bceR* strain (Figure 2D).

### Mitogenicity and Pro-inflammatory Response Induced by GBS in Human PBMCs

**Figure 3:**
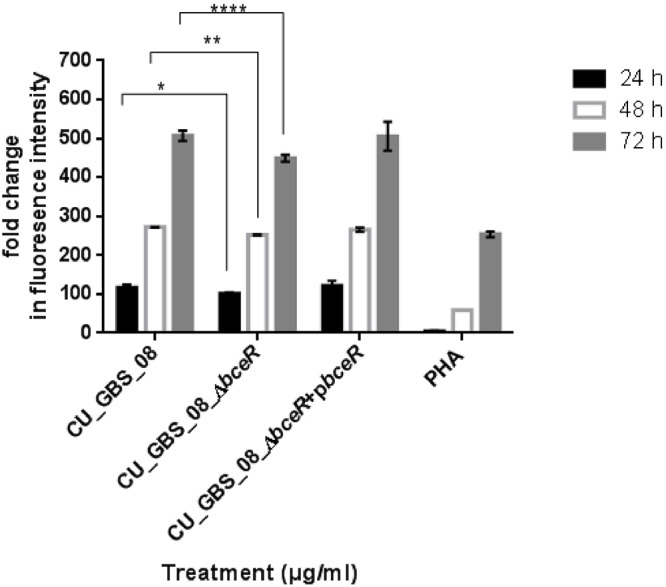
Effect of *bceR* deletion on proliferation of PBMCs. PBMCs were cultured in 25 μg/ml heat-killed GBS strains for 24 h, with phytohaemagglutinin (PHA) (10 μg/ml) only. Cell proliferation was determined by fluorescence intensity. Three independent experiments were performed and the mean ± SD was illustrated with the error bar. Statistical significance at ^∗^*p* < 0.05, ^∗∗^*p* < 0.01, ^∗∗∗∗^*p* < 0.0001 were reached when GBS wild type strain was compared to the mutant (CU_GBS_08_Δ*bceR*). The mitogenicity effect between the wild type and Δ*bceR* strain was most significant at 72 h (*p* < 0.001).

**Figure 4:**
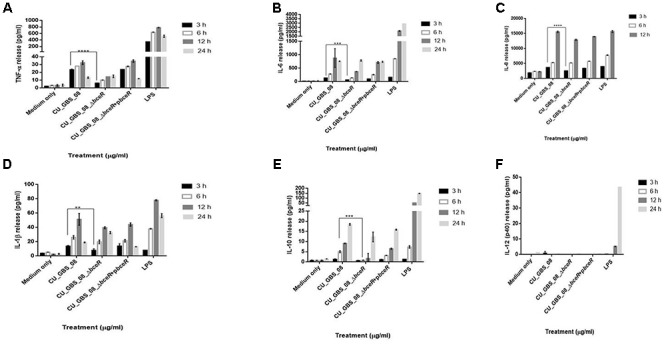
Release of TNF-α, IL-6, IL-8, IL-1β, IL-10, and IL-12 **(A–F)** from human lymphocytes as induced by wild type GBS and Δ*bceR* strain. Heat-killed GBS extract (5 μg/ml) (invasive strain CU_GBS_08; CU_GBS_08_Δ*bceR*; CU_GBS_08_Δ*bceR+pbceR*, *bceR* complementation strain) was incubated with human lymphocytes. Cytokine release was measured at 3, 6, 12, and 24 h. LPS represents the lipopolysaccharide control. Data are expressed as mean ± SD (^∗∗^*p* < 0.01, ^∗∗∗^*p* < 0.001, ^∗∗∗∗^*p* < 0.0001).

The proliferation of PBMCs was evaluated after 24 h of stimulation with GBS or 10 μg/ml PHA to evaluate mitogenicity and the ability of GBS to induce the proliferation of these cells. As shown in Figure 3, although all bacteria induced the proliferation of PBMCs, the Δ*bceR* strain demonstrated a significantly reduced immunogenicity (*p* < 0.0001). Similarly, levels of the cytokines TNF-α, IL-6, IL-8, IL-1β, IL-10, and IL-12 were determined, as shown in Figures 4A–F. The isogenic mutant strain Δ*bceR* induced a significant decrease in the expression of pro-inflammatory cytokines when compared to that with the wild-type strain. The decreased release of TNF-α was the most obvious (*p* < 0.0001) and was approximately fourfold decreased compared to that with the wild-type strain. This was followed by IL-6, IL-1β, and IL-10, which were decreased by approximately twofold with the Δ*bceR* strain (*p* < 0.001 for IL-6 and IL-10 and *p* < 0.01 for IL-1β). Peak IL-6 expression was delayed to 24 h with the Δ*bceR* strain, and the release of IL-8 was approximately 1.4-fold lower for this strain (*p* < 0.0001). The release of IL-12 could not be detected in the presence of both wild-type and mutant strains. Further, the complementation of Δ*bceR* using pDL289 reversed the change in cytokine release.

### The Deletion of *bceR* Attenuates Virulence in a Mouse Infection Model

**Figure 5:**
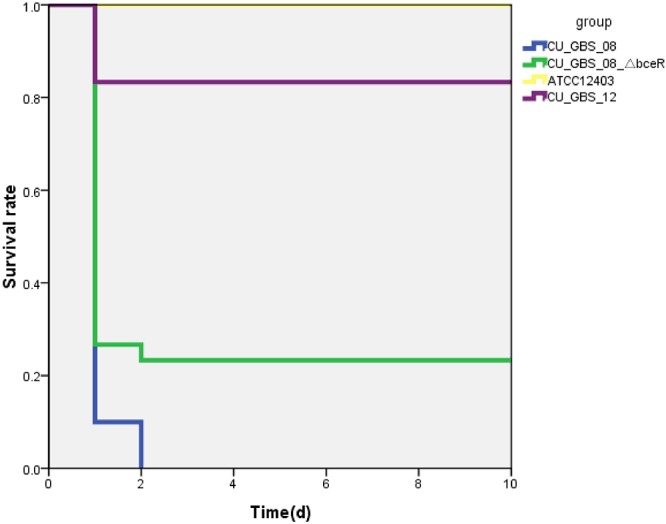
Kaplan–Meier survival curve. Survival rate was calculated at 10 days post-intraperitoneal injection. The difference in survival rate between CU_GBS_08 strain (wild type) and CU_GBS_08_ΔbceR strain is statistically significant with P < 0.01 by Fisher’s exact test.

The virulence of the wild-type and Δ*bceR* strains was studied using a mouse infection model via intraperitoneal inoculation. The lethal concentration (LD_50_) at which 50% of the mice died in the tested group at the specified time point was then calculated. The LD_50_ values of the Δ*bceR* and wild-type strains were 1 × 10^7^ and 3 × 10^6^ CFU, respectively (Supplementary Table S4). Moreover, the survival rates of mice infected intraperitoneally with GBS at 10^7^ CFU after 10 days of inoculation are shown in Figure 5. As observed, the virulence of the Δ*bceR* strain was attenuated compared to that of the wild-type strain, as revealed by the increased survival rate of 23.3% versus 0% with the wild-type strain (*p* < 0.01).

### Bacterial Survival in Response to H_2_O_2_ Stress Is Decreased in the Δ*bceR* Strain

**Figure 6:**
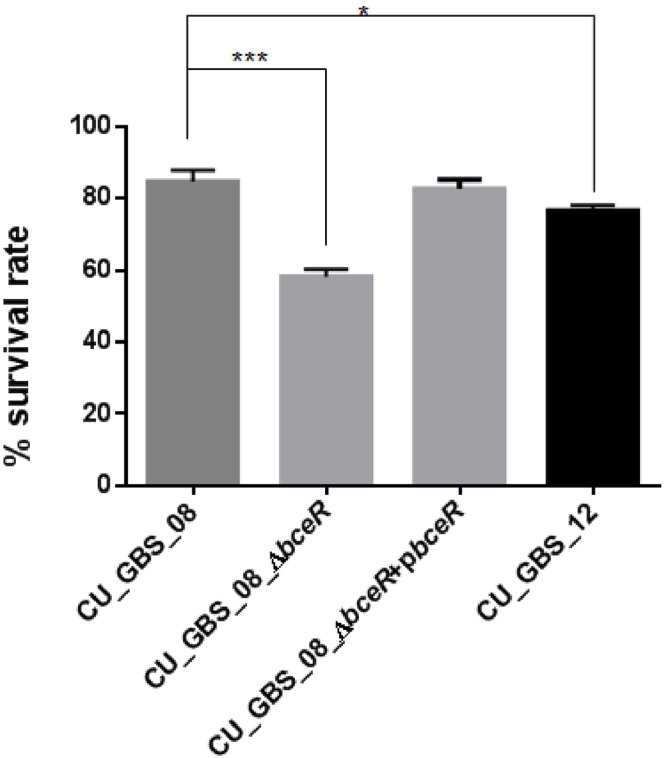
Effect of H_2_O_2_ stress on GBS. The Δ*bceR* was significantly more susceptibility to H_2_O_2_ (40 mM) exposure than wild type (^∗^*p* < 0.05, ^∗∗∗^*p* < 0.001).

Next, the response of the Δ*bceR*, wild-type, and complementation strains to H_2_O_2_ stress was assessed (Figure 6). The mutant strain was significantly more susceptible to H_2_O_2_ than the wild-type strain. Specifically, the survival rate of the mutant strain was reduced by 20% compared to that of the wild-type strain (*p* < 0.001); however, no significant difference in susceptibility was observed between wild-type and non-invasive CU_GBS_12 strains.

### Biofilm Formation Is Impaired in the Δ*bceR* Strain

**Figure 7:**
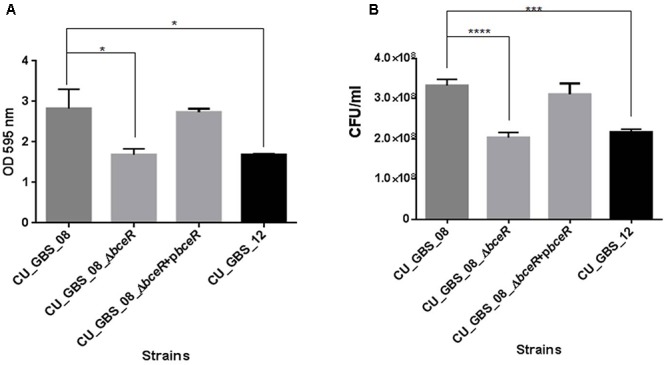
Evaluation the role of two-component regulator *bceR* on biofilm formation in GBS using crystal violet staining **(A)** and bacterial counting **(B)**. Bacteria stained with crystal violet were measured at OD_595_. Significance was determined by one-way ANOVA (^∗^*p* < 0.05, ^∗∗∗^*p* < 0.001, ^∗∗∗∗^*p* < 0.0001).

The ability of the wild-type, Δ*bceR*, Δ*bceR* complementation, and CU_GBS_12 (non-invasive) strains to form biofilms was assessed by crystal violet staining and CFU enumeration (Figures 7A,B). One-way ANOVA analysis showed that biofilm formation was impaired significantly in the Δ*bceR* strain when compared to that in the wild-type strain (*p* < 0.05 and *p* < 0.0001, for crystal violet staining and CFU numbers, respectively), which was reversed by complementation. The biofilms were also evaluated by confocal microscopy (CLSM), wherein the cell density (xy images) and thickness (xz images) of biofilms were assessed. As shown in Supplementary Figures S1A–C, most cells in the biofilms were stained green, indicating that more live cells were present. However, a decreased signal was detected, based on the xy and xz images, for the *bceR* strain when compared to that with the wild-type strain, which indicated that fewer living or dead cells were present with the Δ*bceR* strain. Thus, CLSM images revealed that loss of the *bceR*-like regulator inhibited biofilm formation, resulting in a lower cell density and reduced thickness.

### Deletion of *bceR* Alters Protein Expression in the GBS Strain

**Figure 8:**
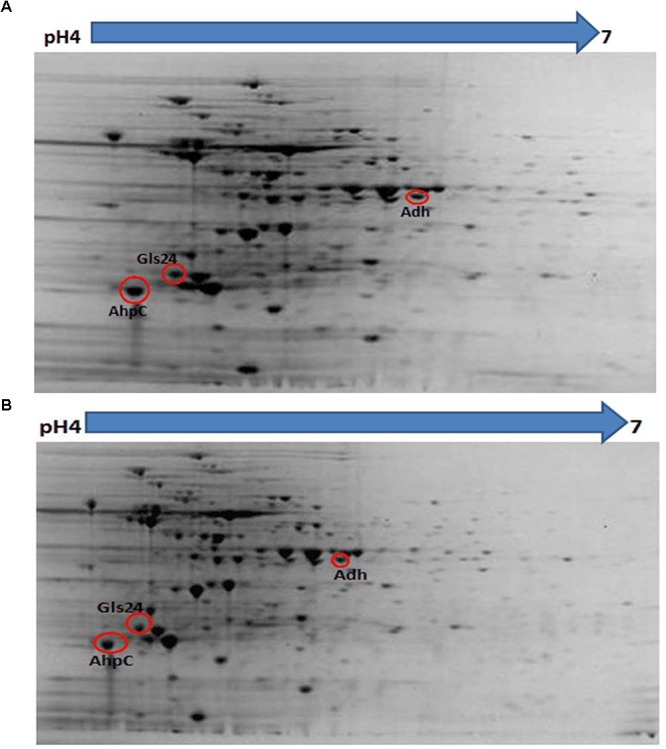
Photographs of 2-DE gel in wild type **(A)** and Δ*bceR* strain **(B)** GBS strain. Red circles showed the proteins that found to have significantly decreased expression in Δ*bceR* strain. They were identified as alkyl hydroperoxide reductase (AhpC), The Gls24 family stress protein (Gls24), and alcohol dehydrogenase (Adh), respectively, by mass spectrometry. The gel photos were normalized and compared using software PDQuest (Version8.0.1, Bio-Rad, United States), Boolean method was chosen for detecting the proteins with statistic significantly difference in expression between GBS III-4 wild type and mutant strains.

**Figure 9:**
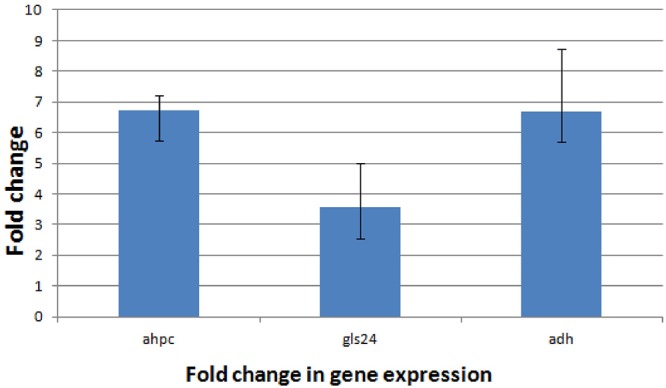
Application of the 2^-ΔΔC_T_^ method. The experiment was conducted to validate the effect of *bceR* gene knockout on the expression of candidate genes. *ahpC*, alkyl hydroperoxide reductase; *gls24*, gls24 family general stress protein; *adh*, zinc-dependent alcohol dehydrogenase. Error bars represent the standard deviation of the mean values from at least three replicate.

Proteomic analysis of bacteria harvested at mid-log phase was performed using 2-DE and mass spectrometry. This revealed three proteins that were reduced by greater than twofold in the Δ*bceR* strain; the Boolean operation of the PDQuest software (version 8.0.1, Bio-Rad, United States) was then used to compare the intensities of the protein spots (Table 3 and Figure 8). This analysis indicated that alkyl hydroperoxide reductase (AhpC), the Gls24 family stress protein (Gls24), and alcohol dehydrogenase (Adh) were decreased by 2.72-, 2.79-, and 2.59-fold, respectively. Real-time PCR was conducted to confirm the results of 2DE-mass spectrometry at the RNA level, and these three markers were reduced by 6.73-, 3.56-, and 6.7-fold, respectively, in the Δ*bceR* strain (Figure 9).

**Table 3:** Results of proteins identification by mass spectrometry.

Spot	Protein name	Protein score	Database	Accession number	Reference
1	Alkyl hydroperoxide reductase (AhpC)	391	NCBInr	gi| 403643060	Cosgrove et al., 2007
2	Gls24 family stress protein (Gls24)	291	NCBInr	gi| 446353446	Teng et al., 2005
3	Zinc-dependent alcohol dehydrogenase (Adh)	409	NCBInr	gi| 446571849	Suvarna et al., 2000; Mukherjee et al., 2006

## Discussion

In this study, the *bceR*-like gene, belonging to the *bceRS*-like TCS family was described in GBS, and was found to mediate AMP and environmental stress resistance. The *bceR*-like system is associated with resistance to cell wall-targeting antimicrobial peptides in *B. subtilis* (Bernard et al., 2007; Dintner et al., 2014). Moreover, the *bceR*-like system (*graR*) of *S. aureus* was previously found to respond to vancomycin and polymyxin B, and the homologous proteins encoded by these genes were determined to mediate resistance to bacitracin and nisin in *S. mutans* and *Lactococcus lactis*, respectively (Tsuda et al., 2002; Kramer et al., 2006). In GBS, we found that the deletion of *bceR* resulted in an increased sensitivity to bacitracin and human cathelicidin LL-37. The regulatory effect of *bceR* on the ABC transporter *bceAB*, which encodes a protein that can pump out AMPs from the bacterial cells, is possibly the major mechanism of AMP resistance conferred by the *bceR*-like system of GBS. However, the loss of *bceR* in GBS did not alter sensitivity to erythromycin and beta-lactam antibiotics; these results demonstrate that the structurally homologous *bceRS* system might play a specific role in GBS, which highlights the importance of determining the individual roles of *bceR*-like systems in the pathogenesis of different Gram-positive species.

In *S. epidermidis*, the TA alanylation system, *dltAB*, and *mprF*, which encodes a lipid modification enzyme, were also found to be controlled by the *bceR*-like system (Li et al., 2007b; Sass et al., 2008). In GBS, the D-alanylation of TA was found to confer resistance to cationic peptides, and the lack of DltA was related to increased sensitivity to phagocytic cells and attenuated bacterial virulence (Poyart et al., 2003; Saar et al., 2012). DltA is a cytoplasmic carrier protein ligase that catalyzes the D-alanylation of the D-alanyl carrier protein DltC. DltB is a transmembrane protein that was reported to be involved in the efflux of activated D-alanine to the site of acylation (Joseph et al., 2004; Mandin et al., 2005). In GBS, we found that the expression of *dltA* was downregulated in the Δ*bceR* strain in the presence of bacitracin, suggesting that it might be regulated by the *bceR*-like system. Suppressing the D-alanylation of lipoteichoic acids through the repression of *dltA* would increase the negative charge of the GBS envelope, resulting in susceptibility of the Δ*bceR* strain to AMPs. In addition to the negative charge of bacteria, the density of the cell wall was shown to be altered in *dltA* mutants of *Streptococcus pyogenes* and the deletion of this gene was found to suppress the production of virulence-related proteins (Cox et al., 2009; Grubaugh et al., 2018; Luo et al., 2018). Moreover, the *bceR*-like system (*virRS*) was identified to regulate bacterial adhesion and entry into eukaryotic cells in *Listeria monocytogenes*, and the *dlt* operon, *mprF*, and *bceAB* were all found to be controlled by the regulator *virR* (Abachin et al., 2002; Camejo et al., 2009), suggesting that *dltA* might contribute to virulence in GBS, which requires further investigation. *mprF* was not differentially expressed in the presence or absence of bacitracin, indicating that this gene might respond to other inducers.

In addition to resistance to AMPs, GBS *bceR* was found to mediate environmental stress resistance and biofilm formation. Accordingly, the Δ*bceR* strain displayed increased sensitivity to H_2_O_2_ stress when compared to the invasive CU_GBS_08 strain, which was similar to results reported for the TCS *graRS* of *S. aureu*s, which was found to be involved in resistance to superoxide radicals (Falord et al., 2011). The underlying mechanism is still unclear, but we found that the Δ*bceR* strain exhibited reduced expression of the alkyl hydroperoxide reductase AhpC, the zinc-dependent alcohol dehydrogenase Adh, and a Gls24 family protein. These proteins have been reported to be involved in oxidative stress resistance and biofilm formation (Becker et al., 2001; Teng et al., 2005; Cosgrove et al., 2007), implying the contribution of the *bceR*-like regulator to these processes in GBS. Experiments demonstrating the effects of other environmental factors on the survival of the wild-type/mutant strains, such as different pH, temperature, and osmotic pressure, were also consistent with results from previous studies on GBS (Yang et al., 2012). However, significant differences in pH tolerance, temperature tolerance, and osmotic stress resistance between wild-type and Δ*bceR* strains were not detected (data not shown). Bacterial cells within biofilms are difficult to eradicate, as they are highly resistant to antibiotics and the host immune system. The difference in biofilm-forming ability between GBS isolates from asymptomatic pregnant women (carriers) and those isolated from clinical infections was previously found to be statistically significant (Olson et al., 2002). The protein Adh was previously reported to catalyze the reversible conversion of acetaldehyde to ethanol, which is known to enhance the production of *Staphylococcus* biofilms; moreover, Adh expression was found to be upregulated in *Staphylococcus* biofilms (Becker et al., 2001; Finelli et al., 2003). In our study, all strains were able to form biofilms, but the biofilm biomass of the wild-type strain was significantly greater than that of the Δ*bceR* strain. This is consistent with a previous report suggesting that the TCS *graRS* is involved in biofilm formation in *S. aureus* (Shanks et al., 2008).

The invasive CU_GBS_08 strain used in this study was isolated from a non-pregnant adult with toxic shock syndrome, indicating the virulence of this invasive clinical strain. Therefore, the role of the *bceR*-like system in virulence was assessed by using both *in vitro* cytokine release assays and an *in vivo* mouse infection model. Our results demonstrated the mitogenic nature of this regulator and its ability to induce a significant pro-inflammatory cytokine response, which is a characteristic of the development of sepsis and septic shock. Cytokines are soluble proteins that play a significant role in inflammation and the regulation of immune responses (von Hunolstein et al., 1997). Significantly increased production of TNF-α, IL-6, and IL-1β was detected after infection with the wild-type strain compared to that with the Δ*bceR* strain. These three cytokines were reported to be positively related to disease severity (De Bont et al., 1993; Cusumano et al., 1996; von Hunolstein et al., 1997). It was previously reported that *S. epidermidis* and *S. aureus* mutant strains devoid of the *bceR*-like system are more susceptible to neutrophil-mediated killing (Cheung et al., 2010). Moreover, the expression of IL-8, a major activator of neutrophils and lymphocytes (Cusumano et al., 1996; Vallejo et al., 1996; von Hunolstein et al., 1997) was found to be reduced in Δ*bceR* strains. However, the deletion of *bceR* did not completely abrogate the proliferation of mononuclear cells and cytokine release, suggesting that other factors are also involved in the virulence and pathogenicity of this strain.

In our mouse infection model, ATCC12403, which originated from a case of fatal septicemia, was used as a control. Our wild-type invasive strain resulted in lethality that was decreased by two orders of magnitude compared to that with the ATCC strain, thus indicating its hyper-virulence. Further the attenuation of virulence in the Δ*bceR* strain was demonstrated; moreover, the Gls24 family protein was previously found to be related to bacterial virulence (Teng et al., 2005). The *bceR*-like system was previously found regulate numerous virulence factors in *S. aureus* and *L. monocytogenes* (Joseph et al., 2004; Falord et al., 2011), which in turn indicates that *bceR* might be involved in cross-talk with other regulator(s) in GBS. The non-invasive GBS strain was the least virulent among the stains tested, and harbors mutations in addition to the *bceR* truncation; this indicates that other gene(s) involved in bacterial virulence need to be characterized. TCSs are widely used as signal transduction systems by bacteria to respond to changing growth conditions. The ability of GBS to efficiently adapt to different host niches during the infectious cycle is important for the pathogenicity of these strains. *bceRS*-like TCSs are widespread in Gram-positive bacteria and are associated with a range of bacterial activities. Further, their contributions to these activities in GBS have not been sufficiently recognized. Our results indicated that *bceR* is involved in environmental stress resistance, antimicrobial peptide resistance, and virulence, processes that are crucial for the survival of GBS in response to different microenvironments that are encountered during infection. Thus, *bceR* could be a potential target to modulate and attenuate virulence.

## Author Contributions

YY, ML, HZ, CL, and AL performed the experimental work. YY analyzed the data with supervision of MI and prepared first draft of the manuscript. MI and KF contributed to the GBS strains collection and design of the project. MI and GZ contributed essential ideas and discussion. All authors contributed to the drafts of the manuscript, revision and approved the manuscript submission.

## Conflict of Interest Statement

The authors declare that the research was conducted in the absence of any commercial or financial relationships that could be construed as a potential conflict of interest.

## Footnotes

1 https://www.ncbi.nlm.nih.gov/genome/genomes/186; accessed Mar2018

2 http://www.geneious.com

3 https://www.atcc.org/products/all/12403.aspx
