# Supplementary material for: Role of Two-Component System Response Regulator bceR in the Antimicrobial Resistance, Virulence, Biofilm Formation, and Stress Response of Group B Streptococcus
Source: Front Microbiol. 2019 Jan 23;10:10. doi: 10.3389/fmicb.2019.00010 (PMC6351488; doi:10.3389/fmicb.2019.00010)
Supplement: Supplementary file 1 [file Data_Sheet_1.docx]

Supplementary Material

Role of two-component system response regulator *bceR* in the antimicrobial resistance, virulence, biofilm formation, and stress response of Group B Streptococcus

Ying Yang, Mingjing Luo, Haokui Zhou, Carmen Li, Alison Luk, GuoPing Zhao, Kitty Fung, Margaret Ip*

Department of Microbiology, The Chinese University of Hong Kong, Shatin, Hong Kong

* Correspondence:
Dr. Margaret Ip
E-mail: [margaretip@cuhk.edu.hk](mailto:margaretip@cuhk.edu.hk)

# Supplementary Tables and Figure

# Supplementary Table 1. List of primers for constructing △*bceR* mutant and complementary strains.

| **Gene target** | **Forward** | **Reverse** | **Purpose** |
| --- | --- | --- | --- |
| *bceR* | 5'-TCAAGGATCCAGAATGATTTCCGTCGTTTAGGT-3' | 5'- CCCCGAATTCCACTAGAGACATGATAGCTAGCT-3' | Flanking the two ends of the genes |
| *bceR* | 5'- CCCCGAATTCCACTTATGACACGCTTACGT-3' | 5'- TCACGGGTACCTATAGAAACAGCAGTCCCATAAT-3' |  |
| *bceR* | 5'- AAAGGGAACAAAAGCTGGAGCTCCAC-3' | 5'- GTTTTCCCAGTCACGACGTTGTAAAACGA-3' | Crossover PCR |
| *bceR* | 5'- AATTCAAAAGGTGACGCGAAAAATATATTTGAAGA-3' | 5'- ATACCCAACTCCTCTCACTGTATGAATGTAATCAA-3' | Checking plasmid integration into the chromosome |
| *bceR* | 5'- AGCAAGGAAAAATTTATATTGTAGAAGATGATATGA-3' | 5'- ATCGTATTGATGCTTCAAGTGTAGAGAAAATGGTT-3' |  |
| *bceR* | 5'- AATTCAAAAGGTGACGCGAAAAATATATTTGAAGA-3' | 5'- ATCGTATTGATGCTTCAAGTGTAGAGAAAATGGTT-3' | Checking plasmid excision from the chromosome |
| *bceR* | 5'- ATATCTAGAGGTGGGTTATTATTTACTGG-3' | 5'- ATAGGATCCGGGCATTGGTAAATGATATAG-3' | Complementation of CU_GBS_08_*△BceR* |

**Supplementary Table 2**. Primers used in real-time PCR.

| **Primer** | **Sequence** |
| --- | --- |
| *bceR_F* | 5'- TGTCAGCAATTTTCGTGATGTGA-3' |
| *bceR_R* | 5'- ACGCAATTCTGCAGTCCAGT-3' |
| *bceA_F* | 5'- CAGCGGCAACGGTTCTTTTT-3' |
| *bceA_R* | 5'- CACGCGCAACTTGACGTTTA-3' |
| *bceB_F* | 5'- AACGTGTAGCGATTGCTCGT-3' |
| *bceB_R* | 5'- ACGGTAATCTAACGCTGCTGT-3' |
| *dltA_ F* | 5'- TTGGTAGGGCAAACAGGGTG-3' |
| *dltA_R* | 5'- TGGCTCAACCGCCATATTCA-3' |
| *mprF_F* | 5'- GCCACCGAATCCTGACAAGT-3' |
| *mprF_R*  *ahpc_F*  *ahpc_R*  adh_F  adh_R  *gLS24_F*  *gLS24_R* | 5'- GTGGGGGTTATCGCTGTCTT-3'  5'- CTGATGGCATCGGACGTGAT-3'  5'- CCTGGATGTTGGCGGATGTA-3'  5'- TGGCTGTGAGTGGAGGTTTC-3'  5'- GCTGGGATATCCTTGCCGTA-3'  5'- CCAGGTACCAGGACGCATTT-3'  5'- GACACGATCACCAACCCTCA-3' |
| *16s rRNA F* | 5'- TGGAGCATGTGGTTTAATTCGA-3' |
| *16s rRNA R* | 5'- TGCGGGACTTAACCCAACA-3' |
|  |  |

**Supplementary Table 3.** List of truncated genes in non-invasive strain (CU_GBS_12) vs. invasive strains**.**

| **Gene** | **Mutations in CU_GBS_12** | **Potential Function of Protein** | **References** |
| --- | --- | --- | --- |
| *bceR* | c.288delG | - Regulates *bceRSAB* operon, required for bacitracin resistance in *Streptococcus mutans* | Dintner et al., 2011 |
| *mecA* | c.8_20del TGAAACAAATCAG | - Negative regulator of competence in *Streptococcus mutans* | Tian et al., 2013 |
| *murA* | c.894delA | - Catalyzes first step of peptidoglycan biosynthesis | Du et al., 2000 |
| *wfgD* | c.110G>A | -Catalyzes second step of polysaccharide synthesis. | Zhang et al., 2016 |

Supplementary Table 4. LD_50_ of GBS mouse infection models.

| **GBS Strain** | **LD_50_^a^** |
| --- | --- |
| CU_GBS_08 | 3*10^6^ |
| CU_GBS_08_△*bceR* | 1*10^7^ |
| CU_GBS_12 | 2*10^7^ |
| ATCC 12403 | 3*10^8^ |

^a^LD_50_ expressed as bacterial c.f.u. (colony-forming units).

LD_50_ calculated by survival rate 10 d post- intraperitoneal injection, using the method of Reed and Muench.

**
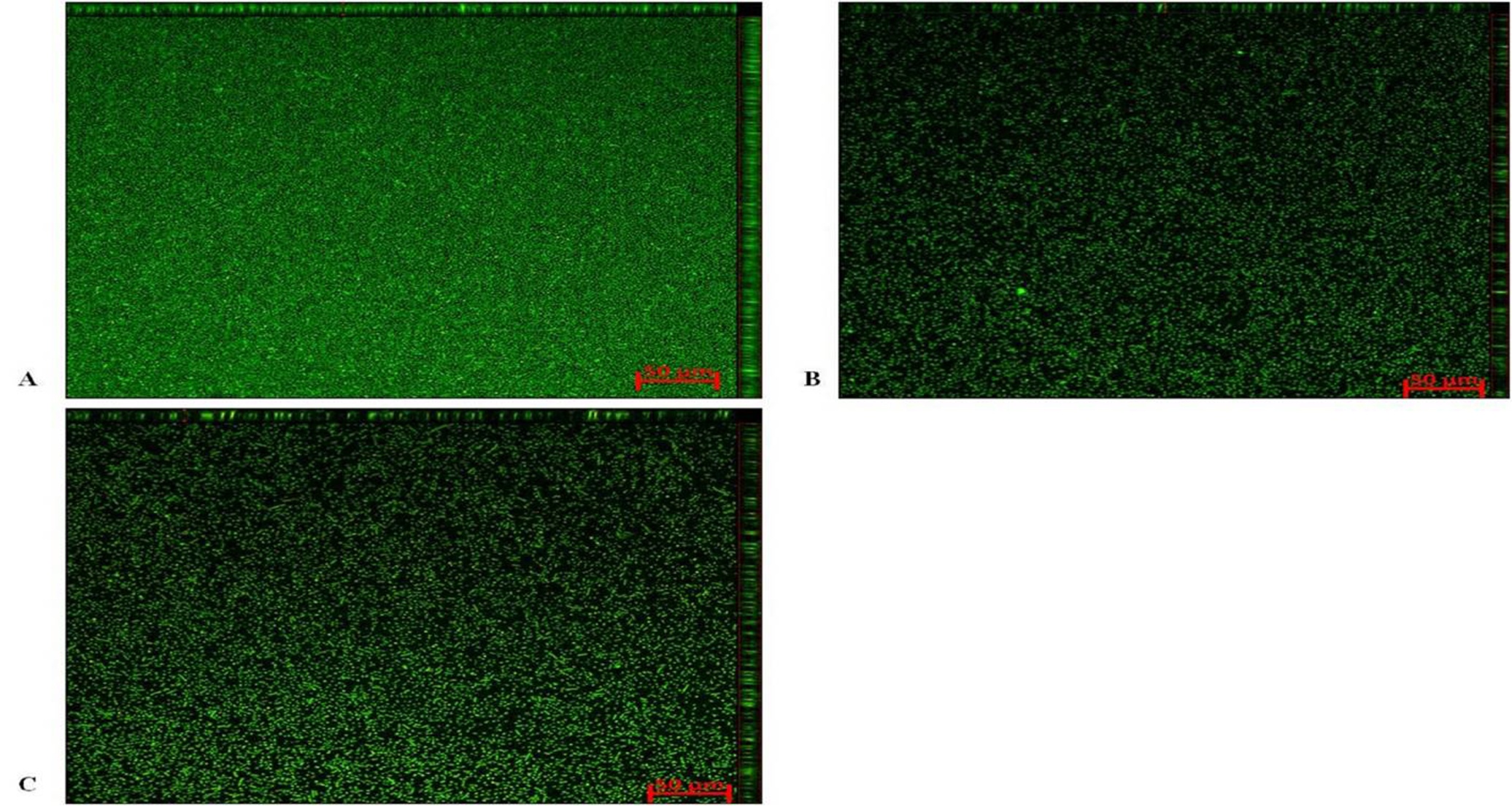
**

**Supplementary Figure 1.** Confocal microscopic images of biofilms in CU_GBS_08 (A), CU_GBS_08_△*bceR* (B), and CU_GBS_12 strains (C), respectively. Biofilm formation was inhibited in △*bceR* strain. Biofilms were stained with BacLight, showing viable (green fluorescence) and nonviable (red fluorescence) GBS bacteria within the biofilms. The assays were performed at least three times.
